# The complete chloroplast genome of *Potaninia mongolica* (Rosaceae) from China

**DOI:** 10.1080/23802359.2019.1710295

**Published:** 2020-01-14

**Authors:** Lin Cong, Haiyan Jiang

**Affiliations:** Forestry College, Inner Mongolia Agricultural University, Huhhot, PR China

**Keywords:** *Potaninia mongolica*, chloroplast genome, next-generation sequencing

## Abstract

The complete chloroplast (cp) genome sequence of *Potaninia mongolica* has been characterized in this study. The length of cp genome was 152,982 bp, containing a large single-copy region (LSC) of 84,233 bp and a small single-copy region (SSC) of 18,139 bp, which were separated by a pair of 25,305 bp inverted repeat regions (IRs). The genome contained 132 genes, including 86 protein-coding genes, 38 tRNA genes, and eight rRNA genes. The overall GC content is 37.16%. Further, phylogenetic analysis suggested that *Potaninia* is clustered to genus *Dasiphora*.

*Potaninia mongolica* Maxim, belonging to single-species Potaninia genus of Rosace family (Li et al. 2003). It is a rare and endangered plant in Alasan desert and has special patterns of drought resistance (Gao et al. 2001). Much attention have focused on the ecology and biology fields. However, there is no complete chloroplast (cp) genome of *P. mongolica* in GenBank database. Here, we first reported the complete cp genome of *P. mongolica* and assessed its phylogenetic position within Rosaceae.

Fresh leaves of *P. mongolica* were sampled from national nature reserve in Western Ordos, Inner Mongolia Province, China (40°03′45″N, 106°55′13″E). Voucher specimens were deposited at the herbarium of Inner Mongolia Agricultural University (Voucher number: SLBHZW210). The genomic DNA was extracted using the modified CTAB method (Doyle 1987) and then sequenced using Illumina-HiSeq 2000 platform (Illumina, San Diego, CA), with a 150 bp paired-end running. NovoPlasty was used to assemble the cp genome (Dierckxsens et al. 2017), with the cp genome of *Rosa odorata* var. *gigantea* (Crépin) Rehder & E. H. Wilson as the reference (Genbank accession no. KF753637) (Yang et al. 2014). We annotated the assembled sequence with GeSeq (Tillich et al. 2017). The annotated cp genome sequence has been submitted to NCBI with an accession number of MN691039.

The complete cp genome of *P. mongolica* is 152,982 base pairs (bp) in length, containing a large single-copy region (LSC) of 84,233 bp, a small single-copy region (SSC) of 18,139 bp, and two inverted repeat regions (IRs) of 25,305 bp. The new sequence possesses 132 genes, including 86 protein-coding genes, eight rRNA genes, and 38 tRNA genes. Among them, four rRNA genes (i.e. *rrn16*, *rrn5*, *rrn4.5*, and *rrn23*), seven protein-coding genes (i.e. *rpl2*, *rpl23*, *ycf2*, *ndhB*, *rps7*, *rps12* and *ycf1*), and seven tRNA genes (i.e. *trnI-CAU*, *trnL-CAA*, *trnV-GAC*, *trnI-GAU*, *trnA-UGC*, *trnR-ACG*, and *trnN-GUU*) occur in double copies. The overall GC-content of the cp genome is 37.16%, while the corresponding values of the LSC, SSC, and IR regions are 35.06, 42.90, and 30.95%, respectively.

To further determine its phylogenetic position, we performed phylogenetic analyses using 30 Rosaceae cp genomes with two *Morus* species as outgroups. After alignment by MAFFT v7 (Katoh and Standley 2013), we found model ‘GTR + I+G’ is the fittest model for phylogenetic construction using jModelTest (Posada 2008). Finally, a Bayesian Inference (BI) phylogenomic tree was performed in MrBayes v3.2.3 (Ronquist and Huelsenbeck 2003). The Markov chain Monte Carlo (MCMC) algorithm was run for 1,000,000 generations with trees sampled every 500 generations. Our results showed that *P. mongolica* is clustered to genus *Dasiphora* (Figure 1).

**Figure 1.**
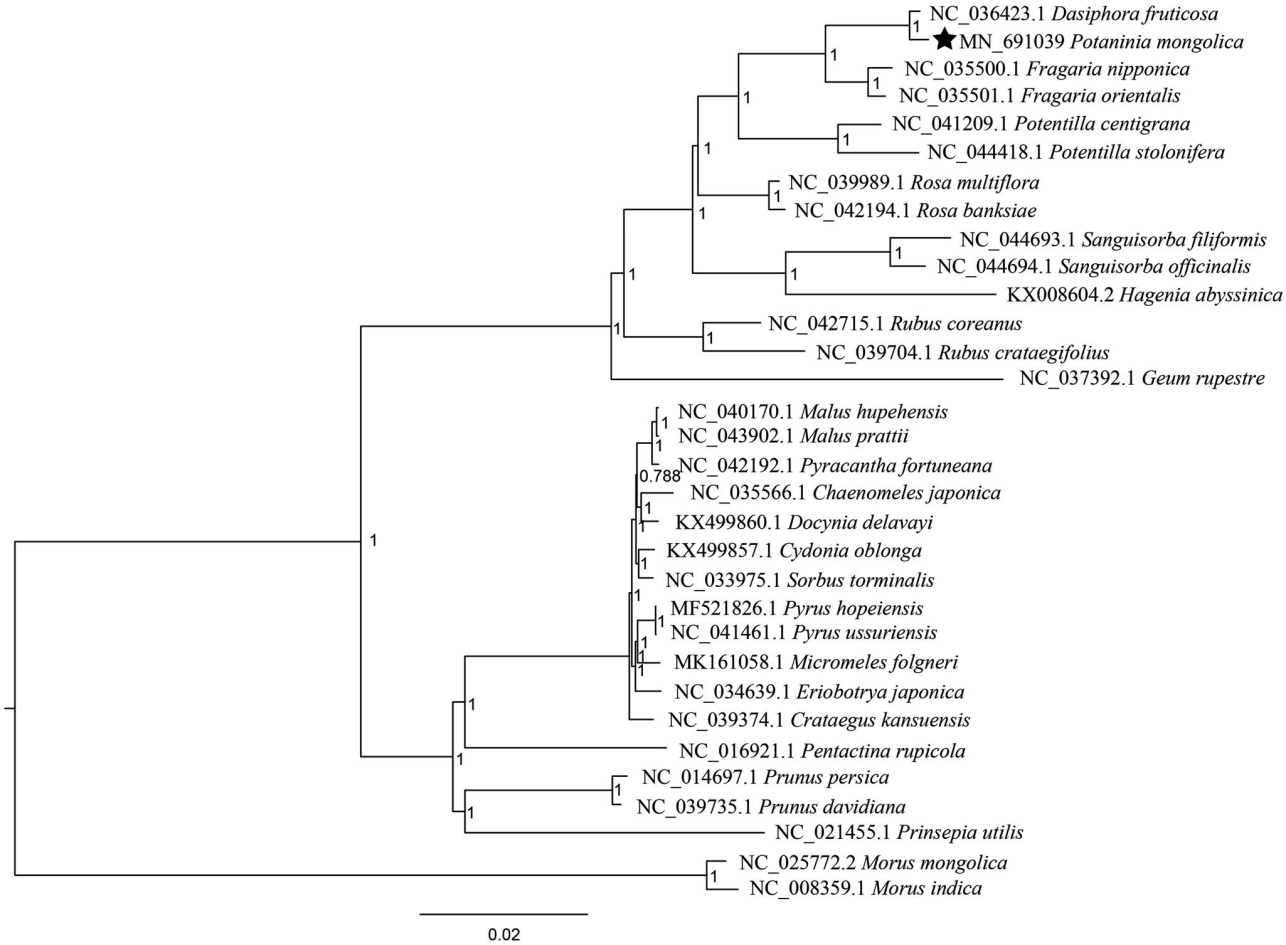
Phylogeny of 30 Rosaceae species based on chloroplast genome sequences. *Morus mongolica* and *M. indica* was selected as outgroups. BI posterior probability is indicated for each branch.

## Geolocation information

The samples in this study were from national nature reserve in Western Ordos, Inner Mongolia province, China (40°03′45″N, 106°55′13″E).
